# Trauma‐informed or sensory‐based practices in preschool settings: A scoping review

**DOI:** 10.1111/1440-1630.70027

**Published:** 2025-05-22

**Authors:** Rebecca Hockey, Kelsey Philpott‐Robinson, Kirsti Haracz, Karen Ray

**Affiliations:** ^1^ School of Health Sciences, College of Health, Medicine and Wellbeing University of Newcastle Callaghan New South Wales Australia; ^2^ Olga Tennison Autism Research Centre, School of Psychology and Public Health La Trobe University Melbourne Victoria Australia

**Keywords:** early childhood education, occupational therapy, sensory environments, trauma‐informed care

## Abstract

**Introduction:**

Trauma is prevalent among preschool children and can significantly impact development, mental health, and engagement in childhood occupations. Evidence suggests trauma affects sensory processing, impacting engagement in everyday activities. Preschool settings may offset the impacts of trauma with social and developmental opportunities. Occupational therapy may provide interventions for trauma in preschools at both organisational and individual levels. Evidence for trauma‐informed (organisational) preschool approaches is limited but growing; however, little is known about the use of sensory‐based (individual) practices. The purpose of this study was to review trauma‐informed and sensory‐based practices in preschool settings, including implementation and evaluation.

**Methods:**

The Preferred Reporting Items for Systematic Reviews and Meta‐Analyses Scoping Review (PRISMA‐ScR) guided the search of six databases for peer‐reviewed primary studies of trauma‐informed and/or sensory‐based practices with children and/or educators in preschool settings.

**Consumer and Community Involvement:**

No consumers were involved in study design or analysis.

**Results:**

Eighteen studies were included in this review (US publications n = 17). Studies involved preschool children (n = 7), preschool educators (n = 8), or both preschoolers and educators (n = 3). Included studies reported on trauma‐informed practices (n = 11), sensory‐based interventions (n = 6), or a combination of both (n = 1). Intervention implementation was multi‐disciplinary; however, occupational therapy was minimally represented. Interventions were for specific durations (defined) or integrated into daily routines (embedded), with defined interventions used predominantly for sensory‐based practices. Interventions were predominantly applied class and/or school‐wide (n = 9). Evaluation encompassed child, staff, and organisational outcomes; however, follow‐up evaluations were infrequent.

**Conclusion:**

Further research on trauma‐informed and/or sensory‐based practices in preschool settings is needed, focussing on implementation methods and long‐term follow‐up evaluations. Limited implementation of interventions by occupational therapists highlights an opportunity to play a more active role in future research, including consultation and individual intervention approaches.

**PLAIN LANGUAGE SUMMARY:**

Trauma can impact how young children process sensory information and significantly affects everyday activities. Preschools play a key role in helping reduce the negative effects of trauma in young children. There is more known about trauma‐informed practices than sensory‐based practices and how they help preschool‐aged children participate in everyday activities. We looked at previous research to find out what is currently known about these supports, who is using them, and how they are being used and evaluated. We followed specific guidelines (the PRISMA‐ScR) to find research articles in six databases. We were interested in articles on trauma‐informed and/or sensory‐based practices for preschool children. We found 18 relevant journal articles, mostly from the United States. Most studies were on trauma‐informed approaches mixed into the daily preschool routine. Fewer studies used sensory‐based interventions, and these were mostly implemented for a set period of time. Most supports were used for an entire classroom or school, but they were rarely evaluated, meaning it remains unclear if the supports worked or not. More research is needed to find out if trauma‐informed and/or sensory‐based practices are helpful for preschool children who have experienced trauma. We suggest that future studies look at how these supports are used and whether they are helpful both in the short and long term. Occupational therapists, who understand how trauma history affects preschoolers' engagement in daily activities, could play a key role in research that looks at developing and evaluating strategies that support participation.

**Key Points for Occupational Therapy**

- Evidence regarding the impact of trauma‐informed and/or sensory‐based occupational therapy practices for preschoolers is lacking.
- As yet, occupational therapy has minimal empirical research on trauma‐informed and sensory‐based practices within preschool settings for children with adverse childhood experiences.
- Evaluation of intervention effectiveness should reflect long‐term impacts of trauma on childhood occupations.

## 1 INTRODUCTION

Childhood trauma is caused by adverse experiences or distress during early development (American Psychiatric Association, 2013). Adverse Childhood Experiences (ACEs) include a broad range of abuse and maltreatment, child neglect, household dysfunction, extreme economic adversity, substance use, loss of a consistent caregiver, or exposure to disasters (Berger et al., 2023; Ozturk & Sar, 2006; Sar, 2011). There is an expanding area of research explaining the link between early childhood trauma and the long‐term impact on psychological health and development in later years (Alisic et al., 2014; Anda et al., 2006; Shonkoff & Phillips, 2000). ACEs can also be linked to negative physical and mental health consequences for children (Graham‐Bermann et al., 2012; Oral et al., 2016) with international studies highlighting young children, specifically from birth to age 6, are at higher risk of exposure to potentially traumatic events relative to older children (Fantuzzo & Fusco, 2007; Chudzik, Corr, & Santos, 2023). Trauma can significantly impact developmental progression in young children, including social, emotional, physical, and cognitive development (Enlow et al., 2012; National Scientific Council on the Developing Child, 2014; Ryan et al., 2017; Wade et al., 2018). Early childhood trauma can therefore be seen to pervasively impact broad occupational therapy domains of concern encompassing play, self‐care, and educational participation (Mason & Stagnitti, 2023), with occupational therapy well placed to work with children impacted by trauma (Lynch et al., 2021).

Preschools (formal educational childcare settings prior to school enrolment) are environments that provide developmentally appropriate educational experiences and social interaction opportunities that may offset the impact of stress on the brain (Bartlett & Smith, 2019; Dinehart et al., 2013) and promote development, learning, and wellbeing (Næsby, 2021). Preschool settings therefore provide an important opportunity to impact the negative effects of ACEs in early childhood (Sun et al., 2024). A significant proportion of children in preschool settings have been found to be affected by childhood trauma (Briggs‐Gowan et al., 2010; Jimenez et al., 2016). Children in preschool settings with a history of exposure to ACEs may exhibit behavioural difficulties (Paolucci et al., 2001), aggression, attention issues, and social challenges (Jimenez et al., 2016). Longer term challenges may contribute to poorer academic outcomes, including difficulties in literacy, language, and math (Jimenez et al., 2016; Jones et al., 2004; Milot et al., 2010; Perfect et al., 2016). Further, repeated trauma has a direct influence on brain development, particularly evident in changes to the sensory cortex (Rinne‐Albers et al., 2013). This can affect the ability to process sensory information, leading to difficulties with self‐regulation (Ogden et al., 2006) and daily functioning (Bar‐Shalita et al., 2008). Occupational therapy's scope of practice includes working within preschool settings to support children's participation and meaningful occupational performance outcomes (American Occupational Therapy Association, 2020). This scope includes collaboration with educators to enhance children's engagement, which may utilise trauma‐informed approaches, and direct support of children who have experienced trauma, commonly utilising sensory‐based strategies (Cahill et al., 2014; Khodarahmi, 2019; Mason & Stagnitti, 2023). However, to date, there has not been an integrated review of trauma‐informed and sensory‐based practices relevant to the occupational therapy scope in preschool settings.

Trauma‐informed practice is a strengths‐based framework, wherein education systems, schools, and staff understand, identify, and effectively address the impact of trauma on students (Quadara & Hunter, 2016). As noted, occupational therapists have an increasing role in early education, which includes direct service provision through therapy sessions with individuals and groups of students, indirect service provision through development and oversight of school‐wide strategies, and capacity building of family and teaching staff to support students (Occupational Therapy Australia, n.d.). A key focus of occupational therapy involvement in education is prevention, early intervention, or remediation of the effects of trauma (OTA, n.d.), aligning with trauma‐informed approaches. Trauma‐informed practices in preschool settings are growing, with a recent review finding a predominant focus on teacher upskilling and teacher‐level outcomes (Sun et al., 2024). However, Sun et al. (2024) also note that, to date, there has been less focus on child, organisational, and caregiver‐level outcomes, indicating that evaluation of effectiveness may need to be viewed through a wider lens. Further, trauma‐informed school interventions primarily focus on older school‐aged children (Loomis, 2018; Purtle, 2020). Given the evidence of the impact of trauma in preschool‐aged children, it is imperative that effective trauma‐informed practices are also available for the preschool population, including those that may be provided or supported by occupational therapists.

Children who have experienced ACEs process trauma‐related stressors through their sensory system (Ogden et al., 2006; Perry, 2009). The processing of external sensory stimuli by the central and peripheral nervous system is known as sensory processing (Dunn, 2014). The brain organises, integrates, synthesises, and incorporates the information to comprehend experiences and generate appropriate responses that are automatic and efficient (Dunn, 2007; Thau et al., 2022). Evidence suggests children with a trauma history are at increased risk for sensory processing deficits, potentially impacting their participation in daily activities (Fraser et al., 2017; Yochman & Pat‐Horenczyk, 2020). Sensory modulation, a crucial aspect of sensory processing, refers to the ability to regulate and generate an appropriate level of responsiveness to sensory input by filtering out unnecessary stimuli and attending to relevant stimuli while sustaining an optimal arousal level (Bar‐Shalita et al., 2005). Children experiencing sensory modulation difficulties may display inappropriate responses to sensory stimuli that their nervous system has perceived as harmful (Mangeot et al., 2001). Furthermore, early childhood trauma can trigger persistent activation of the body's stress response system, causing lasting neurological changes in the brain's stress‐related structures and systems (Bremner, 2003), hindering adaptive responses to the environment (van der Kolk, 2003).

Sensory‐based practices, alongside play, have been found to be the most common interventions used by occupational therapists in Australia and Aotearoa (New Zealand) when working with children who have experienced ACE's (Mason & Stagnitti, 2023). However, the authors note that there is limited empirical evidence for occupational therapy practice with children with complex trauma and call for greater research focus in this area. The research evidence for sensory‐based practices has also been described as limited, but promising, when addressing trauma in children and youth (Fraser et al., 2017). Sensory‐based practices emphasise the importance of sensory experiences in children's development, utilising sensory opportunities and accommodating sensory sensitivities to adapt teaching strategies and environments. Sensory‐based practices have been suggested to support children's capacity to self‐regulate, promote attachment behaviours, and increase participation in daily activities (Da Silva, 2011). Given the significant role of the sensory system in the trauma response and the high prevalence of preschool‐aged children with trauma histories (Briggs‐Gowan et al., 2010; Jimenez et al., 2016), it is important to understand how sensory‐based practices are currently used in preschool environments. Such evaluation is crucial to inform the scope of occupational therapy interventions encompassing both direct and indirect service provision in early childhood education when considering trauma.

Occupational therapists, in alignment with the Occupational Therapy Practice Framework (OTPF‐4), have a broad role in addressing the various factors, including trauma's impacts, that influence a child's participation in daily activities within preschools. This role involves advising trauma‐informed early education, such as supporting relationship building and self‐regulation (Ryan et al., 2017), as well as consulting on embedded preschool‐wide strategies (OTA, n.d.). This is particularly important as preschool educators are recognised as key figures capable of establishing positive and trusting relationships with children (Siegel, 2012), which can be instrumental in supporting young children who have experienced trauma (Bartlett et al., 2017). In addition, occupational therapists also commonly provide sensory‐based practices as part of clinical practice in working with children who have experienced ACEs (Mason & Stagnitti, 2023). For occupational therapists to establish a basis for their work to support both trauma‐informed and sensory‐based practices in preschool settings, it is crucial to gain a comprehensive understanding of current practices and identify opportunities to bridge gaps in service provision and programme development, as well as areas needing further research. Therefore, the objective of this scoping review was to identify and explore current trauma‐informed and sensory‐based practices in preschool settings relevant to the scope of occupational therapy. Given the predominance of sensory‐based practices in complex trauma with children, and the scope of occupational therapy to also provide intervention such as capacity building at an organisational level, it is important to explore the use and outcomes of these varied approaches in the literature.

## 2 METHODS

### 2.1 Positionality statement

Author 1 is a registered occupational therapist. At the time of the study Author 1 was an Honours student at a regional tertiary institution. Author 2 is a post‐doctoral research fellow at Olga Tennison Autism Research Centre, La Trobe University and registered occupational therapist. Author 2's research focusses on child wellbeing, self‐regulation, and supporting neurodiverse students in education settings. Author 3's research is focussed on occupational therapy in mental health, recovery, and the role of lifestyle and health behaviours in people with chronic conditions. Author 4 is a registered occupational therapist with a research focus on early childhood development in educational settings.

### 2.2 Research design

This scoping review was guided by the Preferred Reporting Items for Systematic reviews and Meta‐Analyses extension for Scoping Reviews (PRISMA‐ScR; Tricco et al., 2018), a well‐established methodological framework for conducting scoping reviews. A scoping review design was selected given its utility in answering broad questions in an area of emerging evidence by providing an overview of all available literature (Arksey & O'Malley, 2005). No protocol was registered for this scoping review.

### 2.3 Identifying the research question

A search for existing reviews on the topic was conducted prior to finalising the research question. Whilst no pre‐existing reviews were found during the initial stage, Sun et al. (2024) published their review in 2024, which explored trauma‐informed interventions in early childhood education settings. However, sensory‐based practices in preschool settings were not examined.

The research question was guided by Peters et al. (2020) Population, Concept, and Context (PCC) elements. The context and population were considered as one domain, which included children in the preschool setting, defined as formal educational childcare settings prior to school enrolment. An age range was not specified as entry into formal schooling varies between countries. The review concepts were bifurcated: trauma‐informed and sensory‐based practices. Key concepts were defined, within the context of children in preschool settings, to clarify the focus of the scoping review and guide the search strategy. The concept of trauma‐informed practice refers to an approach that recognises and responds to trauma experienced by children. This involves creating safe and supportive environments and emphasises the need to understand the effects of trauma using trauma‐sensitive practices. The concept of sensory‐based practices refers to an approach that emphasises the importance of sensory experiences in children's development. It includes sensory opportunities, as well as accommodating sensory sensitivities, to adapt teaching strategies and environments. The research question was as follows: What is known from the literature about the use of trauma‐informed or sensory‐based practices in preschool settings? In particular, we were interested in understanding:

1. What types of trauma‐informed or sensory‐based practices are currently being used in preschool settings?
2. How are trauma‐informed or sensory‐based practices being implemented in preschools?
3. How are trauma‐informed or sensory‐based practices being evaluated in preschools?

### 2.4 Inclusion and exclusion criteria

Data sources were included in the review if they met the following criteria: (1) English full text was available; (2) peer‐reviewed; (3) primary research studies; (4) included children and/or educators in a preschool setting; and (5) referred to trauma‐informed and/or sensory‐based practices in the preschool setting. No data restrictions were applied.

### 2.5 Identifying relevant studies

The search strategy was developed in consultation with a university research librarian. Both education and health databases were searched to capture trauma‐informed and sensory‐based practices across a range of disciplines, including psychology, social work, and early childhood education. Occupational therapy was included as a specific search term, as the profession has a body of evidence in sensory practices. An initial limited search of A+ Education and CINAHL Complete databases were undertaken to refine the search strategy.

Database searches were then conducted in CINAHL Complete, ERIC, Scopus, Education Research Complete, PsycINFO, and Medline in March 2023. An example of a specific search strategy used in CINAHL Complete is shown in Table 1.

**TABLE 1:** CINAHL complete search strategy.

Line number	Search term/s
1	Preschool OR Early Childhood Setting OR Early Childhood Centre OR Early Childhood Care OR Early Childhood Education OR pre‐primary OR pre‐k OR pre‐kindergarten OR preparatory OR pre‐nursery OR nursery OR kindergarten OR child care OR childcare OR early childhood OR day care or daycare OR creche OR early learning OR reception OR foundation OR early years OR key stage
2	(MH "Child, Preschool") OR (MH "Schools, Nursery")
3	(MH "Early Childhood Intervention") OR (MH "Early Intervention")
4	(MH "Child Care") OR (MH "Child Day Care")
5	S1 OR S2 OR S3 OR S4
6	trauma informed N4 (practice* OR approach* OR environment*) OR Trauma‐informed N4 (practice* OR approach* OR environment*)
7	(MH "Psychological Trauma")
8	(MH "Pediatric Occupational Therapy")
9	sensory N4 (approach* OR practice* OR environment* OR base*)
10	(MH "Sensory Stimulation") OR (MH "Sensory Motor Integration") OR (MH "Sensory Defensiveness")
11	S6 OR S7 OR S8 OR S9 OR S10
12	S5 AND S11

### 2.6 Study selection

Following the search, all identified citations were exported into EndNote X9 (Clarivate 162 Analytics, PA, USA) and uploaded into Covidence (Covidence, 2023). Title and abstract screening were conducted in Covidence by four reviewers, with all titles and abstracts screened independently by two reviewers. Articles that met the initial inclusion criteria were subjected to a full‐text review by two reviewers. Conflicts at each stage of the screening process were resolved by a third reviewer and team discussions. Prior to data extraction, the first author manually searched reference lists of included studies, and any additional sources underwent the same screening process.

### 2.7 Charting the data/synthesis of results

Data extraction was completed in Covidence. The data extraction template was developed by the first author and finalised in Covidence following team discussions. Data extracted included authorship, year of publication, study location, research methodology, population details (preschool children and/or educators), intervention(s), evaluation method(s), outcome(s), and key findings pertaining to the scoping review question. The data extraction was completed by the first author, with 15% checked by a second reviewer to ensure consistency. Consistent with scoping review methodology, studies were not evaluated for quality (PRISMA‐ScR; Tricco et al., 2018). The *Diagnostic and Statistical Manual of Mental Disorders, Fifth Edition* (DSM‐V) was used to categorise diagnostic groups during data extraction. The data extraction and synthesis process established the scope of trauma‐informed and sensory‐based practices in preschool settings, with the findings being presented in narrative and tabular format to facilitate exploration and understanding.

## 3 RESULTS

**FIGURE 1:**
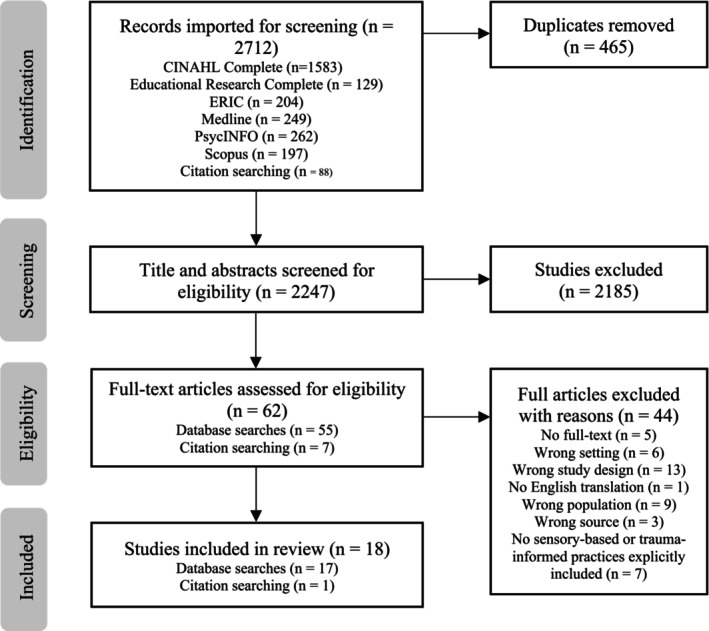
PRISMA‐ScR flowchart.

A total of 2712 studies were identified for screening: 2624 from database searches and an additional 88 from citation searching. After 465 duplicates were removed, a further 2185 studies were excluded by title and abstract screening. Of the 62 remaining studies screened by full text, 44 were excluded as they did not meet eligibility criteria, leaving 18 for data extraction. Figure 1 provides the PRISMA‐ScR flowchart for study selection.

### 3.1 Characteristics of included studies

Of the 18 included studies, 16 were published between 2015 and 2023. Most (n = 17) publications originated from the United States and one originated from Türkiye. The study designs included quasi‐experimental (n = 6), cross‐sectional (n = 3), qualitative (n = 2), mixed‐methods (n = 2), longitudinal (n = 2), ABA single‐subject study/reversal study (n = 1), multiple‐case study (n = 1), and cluster randomised trial (n = 1).

Fourteen studies utilised an experimental approach to investigate interventions, and four were descriptive in nature. Seven studies solely involved preschool children, eight only involved preschool educators, and three included both preschool children and educators as participants. In addition to educator participants, Piller and Pfeiffer (2016) also sought the perspectives of occupational therapists. The lack of homogeneity in participant characteristics for studies with preschool educators hindered the ability to report on this data effectively.

Sample sizes ranged from three to 4442 with a median of 48 participants. Child participants had a mean age of 4 and a half years and an age range of 2 to 6 years and 4 months. Males represented 61.5% of child participants across all studies. Children with a history of trauma or a clinical diagnosis were identified in a small sample of studies (n = 3 and n = 2, respectively). Table 2 outlines further characteristics of included studies. Included studies are marked with an asterisk (*) in the reference list.

**TABLE 2:** Characteristics of included studies.

Characteristics of included studies															
Citation	Study design	Location	Participants			Total sample size (intervention)			Child participant						Aim/s
			Child	Educator	Other	Child	Educator	Other	Mean age (range)	Gender (% male)	Diagnosis			Identified trauma exposure (yes/no)	
											Nil	Autism	IDD		
Bonggat and Hall (2010)	Quasi‐experimental	United States	✓			3			NR (4–4:11)	100%		✓	✓	No	Evaluate effect of sensory integration‐based activities vs. an attention control on on‐task behaviour.
Cerny et al. (2022)	Quasi‐experimental	United States	✓	✓		18	5		NR (3–4)	NR	✓			Yes	Examine effect of occupational therapy group intervention TBRI® Nurture Groups© on social, emotional, and behavioural development.
Chudzik, Corr, and Wolowiec‐Fisher (2023)	Mixed‐methods	United States		✓			25 (survey) 18 (survey and interview)							No	Understand early childhood special education teachers' attitudes and experiences with trauma.
Conners Edge et al. (2022)	Longitudinal	United States		✓			91							N/A	Describe development, implementation and preliminary evaluation of FIRST:ECE. Examine outcomes to establish a foundation for linking programme activities to desired outcomes.
Douglass et al. (2021)	Qualitative	United States		✓			53							N/A	Investigate uptake of trauma‐informed practices in urban early care and education programmes.
Fertel‐Daly et al. (2001)	ABA single‐subject study (reversal)	United States	✓			5			NR (2–4)	60%		✓	✓	No	Examine effectiveness of weighted vests on attention and self‐stimulatory behaviours.
Goldenthal et al. (2023)	Mixed‐methods	United States		✓			13							N/A	Describe development of RLR programme and evaluate its perceived need, feasibility, and acceptability.
Holmes et al. (2015)	Quasi‐experimental	United States	✓			81			4.25 (2:7–6:4)	64%	✓			Yes	Evaluate Head Start Trauma Smart Program.
Lee and Markey (2022)	Cross‐sectional	United States	✓			4442 (2646)			3.4 (NR)	50% (intervention) 49% (control)	✓			Yes	Assess influence of ACEs on children's cognitive, social, and health outcomes, and examine whether Head Start programme mitigates these effects.
Loomis and Felt (2021)	Cross‐sectional	United States		✓			111							N/A	Examine how trauma‐informed training content and attitudes relate to stress levels among teachers and staff.
Loomis et al. (2023)	Cross‐sectional	United States	✓	✓		88	22		NR	52%	✓			No	Examine extent to which trauma‐informed attitudes moderate the association between children's uninhibited behaviour and expulsion risk.
Olson et al. (2016)	Multiple‐case	United States	✓			4			NR (2–5)	75%	✓			No	Examine SPM‐P QT's effectiveness and usability for parents and teachers for implementing intervention addressing sensory processing challenges.
Orapallo et al. (2021)	Longitudinal study	United States		✓			2418							N/A	Evaluate effectiveness of Trauma Smart staff training in, particularly on satisfaction, knowledge and attitudes.
Paul et al. (2003)	Quasi‐experimental	United States	✓			31			4.27 (NR) (total sample) 4.24 (NR) (intervention) 4.3 (NR) (control)	42% (total sample) 47% (intervention) 37.5% (control	✓			No	Evaluate effectiveness of the SITP in treating children with pre‐primary impairments.
Piller and Pfeiffer (2016)	Qualitative	United States		✓	✓		8	5 (occupational therapist)						N/A	Examine viewpoint of teachers and occupational therapists on the sensory‐related environmental barriers to participation.
Shamblin et al. (2016)	Quasi‐experimental	United States	✓	✓		767 (217)	39 (11)		NR	NR	✓			No	Describe combined efforts of Partnerships Program for Early Childhood Mental Health and Project LAUNCH and assess programme's trauma‐informed training on teacher confidence, addressing child behaviours, negative environment attributes and child resilience.
Whitaker et al. (2019)	Cluster randomised trial	United States		✓			96 (48)							N/A	Determine if Enhancing Trauma Awareness, professional development course, improved the quality of teacher–child relationships.
Yeterge et al. (2019)	Quasi‐experimental	Türkiye	✓			34			5.32 (experimental) 5.38 (control)	NR	✓			No	Investigate differences in visual perception and self‐regulation skills with creative sensory integration drama sessions.

*Note*: ACEs = Adverse Childhood Experiences; FIRST:ECE = Fostering Informed and Responsive Systems for Trauma in Early Care and Education; LAUNCH = Linking Actions for Unmet Needs in Children's Health; RLR = Ready to Learn through Relationships; SITP = Sensory Integrative Treatment Protocol; SPM‐P QT = Sensory Processing Measure‐Preschool Quick Tips; TBRI® = Trust Based Relational Intervention®.

### 3.2 Trauma‐informed or sensory‐based practices

Most studies reported on either trauma‐informed (n = 11) or sensory‐based (n = 6) practices, with one study combining both approaches.

Cerny et al. (2022) explored trauma‐informed teacher training and child‐focussed group activities aimed at promoting self‐regulation and social–emotional skills for children with known trauma histories. Both Conners Edge et al. (2022) and Douglass et al. (2021) centred on trauma‐informed teacher training, self‐reflection, classroom strategies, and the implementation of organisational change strategies, including adaptation of policies and procedures. Holmes et al. (2015) targeted children with known trauma histories and consisted of intensive individual trauma‐informed interventions and classroom consultations by a therapist. Staff also received training and peer‐based mentoring in trauma‐informed practice; however, these were not directly measured (Holmes et al., 2015). Lee and Markey (2022) implemented trauma‐informed classroom strategies aimed at promoting school readiness in economically disadvantaged children with a history of trauma. Orapallo et al. (2021) specifically focussed on trauma‐informed training, whereas Shamblin et al. (2016) targeted school systems, with emphasis on trauma‐informed training and classroom strategies. Whitaker et al. (2019) also implemented a training course, enhancing preschool educators' understanding of contemporary trauma and recovery theories.

Three descriptive studies sought to understand educators' perspectives on trauma‐informed practices, and the impact of this approach on other variables. Chudzik, Corr, and Wolowiec‐Fisher (2023) examined special education teachers' attitudes and experiences with trauma‐informed care, whereas Loomis and Felt (2021) discussed the link between teacher training content and educators' attitudes and stress levels. Additionally, Loomis et al. (2023) explored the association between teachers' attitudes and children's inhibition and expulsion risk.

Six studies described sensory‐based practices. None of these studies had participants with identified trauma history. Two studies included children with diagnoses of intellectual developmental disability (IDD) and autism (Bonggat & Hall, 2010; Fertel‐Daly et al., 2001). Fertel‐Daly et al. (2001) was the sole study that examined the use of sensory equipment, with a focus on weighted vests. The remaining five studies examined sensory integration protocols. Bonggat and Hall (2010) compared Sensory Integration‐Based Intervention with attention control activities. Olson et al. (2016) examined Sensory Processing Measure‐Preschool Quick Tips, Paul et al. (2003) investigated the Sensory Integrative Treatment Protocol, and Yeterge et al. (2019) explored a Creative Drama‐Based Sensory Integration Training Program. Piller and Pfeiffer (2016) interviewed preschool teachers and occupational therapists to understand the impact of the sensory environment, including physical and temporal components, on the participation patterns of autistic children.

One study described the combination of sensory‐based and trauma‐informed practices. Goldenthal et al. (2023) described the development of a trauma‐informed programme, encompassing sensory‐sensitive environments and regulation‐based strategies, while also assessing the programme's perceived necessity, feasibility, and acceptability. Table 3 provides further details on practices found in included studies.

**TABLE 3:** Trauma‐informed or sensory‐based practices in preschool settings described in included studies.

Sensory or trauma‐based practices																
Citation	Participants			Type of practice		Intervention implemented (yes/no)	Implemented by	Programme	Practice types				Implementation		Intervention provision	
	Child (C)	Educator (E)	Other	Sensory	Trauma				Sensory‐based strategies	Teacher training	Teacher trauma‐informed reflection	Trauma informed classroom strategies	Individual	Universal	Defined (number of sessions, duration [min], frequency)	Embedded (duration)
Bonggat and Hall (2010)	✓			✓		Yes	Educators		✓				✓	✓	NR, 10 min, NR	
Cerny et al. (2022)	✓	✓			✓	Yes	Educators, occupational therapists	Trust Based Relational Intervention® Nurture Groups©		✓		✓		✓	12, 30 min, weekly (C) NR, NR, NR (E)	
Chudzik, Corr, and Wolowiec‐Fisher (2023)		✓			✓	No				✓		✓				
Conners Edge et al. (2022)		✓			✓	Yes	Educators, external programme facilitators	Fostering Informed and Responsive Systems for Trauma: Early Care and Education (FIRST:ECE)		✓	✓	✓		✓		2 years
Douglass et al. (2021)		✓			✓	Yes	Educators, external programme facilitators	Breakthrough Series Collaborative (BSC)		✓	✓	✓	✓	✓		18 months
Fertel‐Daly et al. (2001)	✓			✓		Yes	Educators		✓					✓	6, 120 min. 3 times a week	
Goldenthal et al. (2023)		✓		✓	✓	Yes	Educators, external programme facilitators	Ready to Learn through Relationships (RLR)	✓	✓	✓	✓		✓		1 year
Holmes et al. (2015)	✓				✓	Yes	Educators, external programme facilitators	Head Start Trauma Smart (HSTS)				✓	✓	✓	12–24, 30–45 min, weekly (individual intervention) NR, 360 min, monthly (classroom consultation)	
Lee and Markey (2022)	✓				✓	Yes	Educators	Head start				✓		✓		1 year
Loomis and Felt (2021)		✓			✓	No				✓	✓	✓				
Loomis et al. (2023)	✓	✓			✓	No										
Olson et al. (2016)	✓			✓		Yes	Educators, occupational therapists, caregivers		✓				✓			3 ½ months
Orapallo et al. (2021)		✓			✓	Yes	External programme facilitators	Trauma Smart staff training		✓				✓	10, 120 min, monthly (in academic year)	
Paul et al. (2003)	✓			✓		Yes	Educators, occupational therapists, external programme facilitators		✓					✓	60, 60 min, daily	
Piller and Pfeiffer (2016)		✓	✓	✓		No			✓							
Shamblin et al. (2016)	✓	✓			✓	Yes	External programme facilitators	Partnerships Program for Project LAUNCH & Early Childhood Mental Health Consultation (ECMHC)		✓		✓		✓		1 year
Whitaker et al. (2019)		✓			✓	Yes	External programme facilitators			✓		✓		✓	6, 150 min, fortnightly	
Yeterge et al. (2019)	✓			✓		Yes	External programme facilitators		✓					✓	10, 45–50 min, 2 times a week	

*Note*: Caregivers = parents or guardians of preschool children; educators = teachers and staff, within preschool settings; external programme facilitators = experts with specialised training, academics, and/or other health professionals, such as psychologists, mental health consultants, physiotherapists, and/or speech therapists, who delivered programmes and/or training to preschools; occupational therapists = health professionals who collaborate within preschool settings to support children's development and participation in daily activities, play, and learning.

### 3.3 Trauma‐informed or sensory‐based practice implementation

Fourteen studies implemented an intervention; four were implemented by educators and external programme facilitators (Conners Edge et al., 2022; Douglass et al., 2021; Goldenthal et al., 2023; Holmes et al., 2015), four were implemented by external programme facilitators only (Orapallo et al., 2021; Shamblin et al., 2016; Whitaker et al., 2019; Yeterge et al., 2019), three were implemented by educators only (Bonggat & Hall, 2010; Fertel‐Daly et al., 2001; Lee & Markey, 2022), one was implemented by educators and occupational therapists (Cerny et al., 2022), one was implemented by educators, occupational therapists, and caregivers (Olson et al., 2016), and one was implemented by educators, occupational therapists, and external programme facilitators (Paul et al., 2003).

Eleven studies described trauma‐informed practices; four implemented a specific intervention across a defined time period (Cerny et al., 2022; Holmes et al., 2015; Orapallo et al., 2021; Whitaker et al., 2019), and four embedded changes to routines and practices throughout the school day (Chudzik, Corr, & Wolowiec‐Fisher, 2023; Conners Edge et al., 2022;Lee & Markey, 2022; Shamblin et al., 2016).

Orapallo et al. (2021) and Whitaker et al. (2019) examined the impact of defined interventions for educators in which sessions lasted an average of 135 minutes and ranged from six to 10 sessions on a fortnightly or monthly basis. For the two studies involving child participants, the number of sessions ranged from 12 to 24, lasting an average of 30 minutes, and were conducted weekly (Cerny et al., 2022; Holmes et al., 2015).

The duration of embedded interventions ranged from 1 to 2 years. Of these studies, two delivered intervention for preschool educators (Chudzik, Corr, & Wolowiec‐Fisher, 2023; Conners Edge et al., 2022), one study for preschool children (Lee & Markey, 2022), and the final study for both preschool educators and children (Shamblin et al., 2016).

We further examined whether studies adopted a universal or individual approach to intervention. We defined universal implementation as practices being applied with a top‐down approach to the entire class or school, whereas individual implementation focusses on interventions with a bottom‐up approach tailored for specific individuals. A universal approach was adopted in six studies, whereas two studies incorporated both individual and universal elements. The remaining three studies about trauma‐informed practices did not implement an intervention. In studies involving preschool children, universal approaches encompassed either whole‐class approaches (Cerny et al., 2022; Holmes et al., 2015; Lee & Markey, 2022) or a hybrid approach combining both whole‐class and whole‐school approaches (Shamblin et al., 2016).

Among the six studies detailing sensory‐based practices, five studies included child participants. Four of the five studies used a defined intervention, with the remaining intervention being embedded over a three‐and‐a‐half‐month period (Olson et al., 2016). Session numbers in the defined interventions varied from six to 60, with an average duration of 60 minutes and frequency from daily to twice weekly. Piller and Pfeiffer (2016) did not involve the implementation of an intervention.

Of the six studies that described sensory‐based practices, three studies explored universal practices with the whole class (Fertel‐Daly et al., 2001; Paul et al., 2003; Yeterge et al., 2019), one study focussed on individual implementation (Olson et al., 2016), and one study used both individual (sensory diets) and universal, whole class elements (Bonggat & Hall, 2010). One study did not implement any intervention (Piller & Pfeiffer, 2016).

The sole study combining trauma‐informed practices and sensory‐based practices utilised an embedded approach across 1 year for preschool educators, in conjunction with a universal approach (whole of class and whole of school) (Goldenthal et al., 2023).

### 3.4 Evaluation of trauma‐informed or sensory‐based practices

The 10 studies with child participants explored outcomes, including child behaviour (n = 10), child development (n = 4), and classroom relationships or environments (n = 5). Teacher outcomes examined included teacher attitudes, knowledge, or skills (n = 9) or teacher stress or resilience (n = 2). At a systemic level, organisational change (n = 2) and programme feasibility and satisfaction (n = 5) were also evaluated.

Of the 11 studies exploring trauma‐informed practices, child behaviour was examined in five studies, conceptualised as regulation and prosocial behaviours (Cerny et al., 2022); attention, externalising problems, and oppositional defiance (Holmes et al., 2015); socioemotional behaviours (Lee & Markey, 2022; Shamblin et al., 2016); and inhibitory control in relation to expulsion risk (Loomis et al., 2023). Two studies evaluated child development, including social–emotional development (Cerny et al., 2022) and cognitive skills such as problem‐solving, receptive language, and social interaction (Lee & Markey, 2022).

Five studies evaluated classroom relationships and environments. Key outcomes included teacher–child relationships (Cerny et al., 2022; Holmes et al., 2015; Loomis et al., 2023; Shamblin et al., 2016; Whitaker et al., 2019) and conflict (Whitaker et al., 2019). At the teacher level, eight studies evaluated teacher attitudes, knowledge, and/or skills. These included knowledge and attitudes (Cerny et al., 2022; Conners Edge et al., 2022; Douglass et al., 2021; Loomis & Felt, 2021; Orapallo et al., 2021) and teaching strategies and practices (Cerny et al., 2022; Conners Edge et al., 2022; Douglass et al., 2021; Loomis & Felt, 2021; Shamblin et al., 2016). Douglass et al. (2021) explored teacher awareness, understanding, empathy, confidence, and empowerment. Shamblin et al. (2016) examined teacher confidence and competence, whereas Chudzik, Corr, and Wolowiec‐Fisher (2023) focussed on educators' experiences with trauma and self‐perceived resilience. Loomis and Felt (2021) explored teacher knowledge, self‐reflection, and attitudes, whereas Loomis et al. (2023) assessed trauma‐informed attitudes.

All outcomes focussed on educator stress and resilience were in studies with a trauma‐informed focus. Chudzik, Corr, and Wolowiec‐Fisher (2023) described educator self‐perceived resilience, and Loomis and Felt (2021) explored general and child‐related stress. All organisational change reported outcomes were in studies with a trauma‐informed focus. Conners Edge et al. (2022) explored the capacity of educators and administrators to support organisational change, whereas Douglass et al. (2021) reported on workplace relationships and culture and interagency collaboration. Three studies looked at specific outcomes for their implemented programmes. Specifically, Orapallo et al. (2021) examined educator satisfaction, Shamblin et al. (2016) explored educators' relationships with programme consultants, and Whitaker et al. (2019) examined fidelity of intervention implementation.

Of the six studies investigating sensory‐based practices, child behaviour was examined in five studies, conceptualised as attention (Bonggat & Hall, 2010; Fertel‐Daly et al., 2001; Yeterge et al., 2019), disruptive behaviours (Bonggat & Hall, 2010), self‐stimulation (Fertel‐Daly et al., 2001), sensory processing across contexts (Olson et al., 2016), sensory integration dysfunction (Paul et al., 2003), and regulation (Yeterge et al., 2019). Child development was evaluated in two sensory‐based studies. Paul et al. (2003) investigated preschool performance, and Yeterge et al. (2019) examined visual perception levels. At the teacher level, Piller and Pfeiffer (2016) looked at the perceptions of preschool educators and occupational therapists regarding sensory aspects of the environment and child participation. Lastly, Yeterge et al. (2019) explored the programme's effects and gathered opinions from children, teachers, and families regarding its impact.

The sole study that examined both sensory‐based and trauma‐informed approaches evaluated programme‐specific outcomes, such as feasibility (Goldenthal et al., 2023).

Ten studies conducted pre‐intervention evaluations (n = 5 sensory‐based and n = 5 trauma‐informed approaches). Post‐intervention evaluations were conducted in 12 studies, and just over half of the intervention studies (n = 7) conducted ongoing evaluation throughout the intervention. Two studies conducted longer term follow‐up evaluations, one sensory‐based study evaluated self‐regulation and the Frostig Visual Perception Test occurring at 4 weeks post intervention (Yeterge et al., 2019), and one trauma‐informed study re‐implemented their educator survey at 5 months post intervention (Whitaker et al., 2019). See Table 4 for further details on evaluation of practices in included studies.

**TABLE 4:** Methods of evaluation identified in included studies.

Citation	Participants			Outcomes							Outcome measure/s	Evaluation conducted		
	Child	Educator	Other	Child behaviour	Child development	Classroom relationships/environment	Educator attitudes, knowledge or skills	Educator stress/resilience	Organisational change	Programme feasibility/acceptability/effectiveness		During	Post	Follow up
Bonggat and Hall (2010)	✓			✓							Nil	✓		
Cerny et al. (2022)	✓	✓		✓	✓	✓	✓				Strengths and Difficulties Questionnaire Teaching Strategies GOLD®;		✓	
Chudzik, Corr, and Wolowiec‐Fisher (2023)		✓					✓	✓			Attitudes Related to Trauma‐Informed Care			
Conners Edge et al. (2022)		✓					✓		✓		Nil	✓	✓	
Douglass et al. (2021)		✓					✓		✓		Nil	✓	✓	
Fertel‐Daly et al. (2001)	✓			✓							Nil	✓	✓	
Goldenthal et al. (2023)		✓								✓	Nil		✓	
Holmes et al. (2015)	✓			✓		✓					Classroom Assessment Scoring System Childhood Trust Events Survey: Caregiver Version Achenbach's Teacher Report Form Child Behaviour Checklist	✓	✓	
Lee and Markey (2022)	✓			✓	✓						Woodcock‐Johnson Tests of Achievement, III, Applied Problems Peabody Picture Vocabulary Test, III	NR	NR	NR
Loomis and Felt (2021)		✓					✓	✓			Attitudes Related to Trauma‐Informed Care Single‐Item Stress Question Preschool Expulsion Risk Measure			
Loomis et al. (2023)	✓	✓		✓		✓	✓				Preschool Expulsion Risk Measure Child Behavior Questionnaire‐Short Form Attitudes Related to Trauma‐Informed Care Student‐Teacher Relationship Scale			
Olson et al. (2016)	✓			✓							Sensory Processing Measure‐Preschool Sensory Processing Measure‐Preschool Quick Tips Record Form	✓	✓	
Orapallo et al. (2021)		✓					✓			✓	Attitudes Related to Trauma‐Informed Care	✓	✓	
Paul et al. (2003)	✓			✓	✓						DeGangi‐Berk Test of Sensory Integration Miller Assessment for Preschoolers		✓	
Piller and Pfeiffer (2016)		✓	✓				✓				Nil			
Shamblin et al. (2016)	✓	✓		✓		✓	✓			✓	Teacher Opinion Scale Preschool Mental Health Climate Scale Devereux Early Childhood Assessment Georgetown University Early Childhood Mental Health Consultation Satisfaction Survey		✓	
Whitaker et al. (2019)		✓				✓				✓	Nil		✓	5 months
Yeterge et al. (2019)	✓			✓	✓					✓	Frostig Developmental Test of Visual Perception		✓	4 weeks

## 4 DISCUSSION

The aim of this scoping review was to identify and explore current trauma‐informed and sensory‐based practices in preschool settings. More specifically, the review described current practices and how they are implemented and evaluated. Eighteen studies met the inclusion criteria for this review, and although limited, a range of trauma‐informed and/or sensory‐based practices were found. Eleven studies examined trauma‐informed practices, six focussed on sensory‐based practices, and one study explored a combination of both. Interventions were implemented by educators (teachers and staff, within preschool settings), occupational therapists (health professionals who collaborate within preschool settings to support children's development and participation in daily activities, play, and learning), external programme facilitators (experts with specialised training, academics, and/or other health professionals, such as psychologists, mental health consultants, physiotherapists, and/or speech therapists, who delivered programmes and/or training to preschools), caregivers (parents or guardians of preschool children), or various combinations of these roles. Practices adopted either interventions with specific durations (defined) or integrated into daily routines (embedded), with sensory‐based practices often utilising a defined approach. Most interventions were applied universally at a whole class or school level. A range of evaluation methods were employed, considering child, staff, and organisational levels, though follow‐up assessments were conducted infrequently. These findings highlight a lack of evidence for trauma‐informed and sensory‐based practices in preschool settings. A significant gap exists, presenting an opportunity to explore more effective implementation and evaluation of these practices and their impact on preschool children's development and well‐being.

We found a small number (n = 11) of studies that explored trauma‐informed practices in the preschool population. Literature suggests that trauma‐informed practices are primarily focussed on older‐school‐aged populations (Loomis, 2018; Purtle, 2020), which is of particular interest given the high prevalence of trauma among preschool‐aged children (Briggs‐Gowan et al., 2010; Jimenez et al., 2016) and the significant negative effects trauma has on child development (Chan & Yeung, 2009; Enlow et al., 2012) and well‐being (Graham‐Bermann et al., 2012; Oral et al., 2016). Furthermore, the review determined an even smaller number of studies examining sensory‐based practices and the combination of both practices. As established, children with ACEs process trauma‐related stressors through their sensory system (Perry, 2009). The link between sensory features and trauma is reflected in direct sensory cortex changes (Rinne‐Albers et al., 2013) and related difficulties in sensory processing and daily functioning (Ogden et al., 2006; van der Kolk, 2003; Yochman & Pat‐Horenczyk, 2020). Additionally, Fraser et al. (2017) suggested limited but promising evidence for the effectiveness of sensory‐based practices within an integrated approach to trauma‐informed practice. However, the limited evidence on trauma‐informed and sensory‐based practices effectiveness in preschools impedes evidence‐based decision‐making for occupational therapists in these settings. Trauma‐informed practice falls within the scope of occupational therapy, and occupational therapists commonly utilise sensory‐based practices in working with children with complex trauma (Mason & Stagnitti, 2023). As such, the profession is uniquely positioned to investigate both direct intervention and preschool‐wide strategies, as well as those that build capacity by providing coaching to upskill families and teaching staff to support preschool‐aged children (OTA, n.d.).

Occupational therapists were found to rarely contribute to the interventions described in the studies included in this review. This is despite many interventions aligning with the core expertise of occupational therapy, and previous reviews and research identifying the role of occupational therapy in working with children who have experienced ACEs. Occupational therapists were more involved in sensory‐based practices (n = 2), compared to trauma‐informed practices (n = 1). Occupational therapists may be able to contribute valuable knowledge of sensory modalities and tools, providing critical insight into the rationale behind the implementation of these interventions. Their consultative role is particularly valuable in supporting preschool educators to integrate appropriate strategies into everyday interactions and ensuring they are informed by a comprehensive understanding of trauma, development, and the unique needs of preschool children. There is a significant opportunity for greater collaboration between occupational therapists and educators, ensuring interventions are effective and evidence‐based.

The included studies demonstrated diverse defined (specific interventions conducted across a designated time period) versus embedded (changes to routines and practices throughout the preschool day) interventions in the implementation of trauma‐informed and/or sensory‐based practices. Trauma‐informed practices were evenly distributed between defined and embedded interventions, whereas sensory‐based practices were more frequently associated with defined interventions. However, literature suggests trauma‐informed practices should be implemented in an embedded and sustained manner due to the long‐term impacts of trauma (Alisic et al., 2014), recognising the effects of trauma and the ongoing need to support young children (Loomis, 2018). There is an opportunity for occupational therapy to contribute to primary research in this area, to support an evidence‐based role in collaborating with preschool settings to support embedded approaches that may facilitate children's inclusion and participation (Campbell et al., 2023). Expanding research for embedded practices will enable occupational therapy to play a proactive role in supporting children's development and participation in preschool settings. Sensory‐based practices, integrated within a trauma‐informed framework, may either focus on the individual, aim for environmental modifications, or leverage occupation or activities to address related sensory processing difficulties (Fraser et al., 2017.) Further research is needed to establish the most effective way to address the well‐documented impacts of trauma on sensory systems and processing. Occupational therapy scope of practice includes children's participation in preschool occupations (Jasmin et al., 2017) and further research in this area may contribute to understanding and addressing the impact trauma has on occupational performance (Snedden, 2012).

Findings also indicated that universal (whole‐class or organisational) interventions in the included studies were commonly adopted across both trauma‐informed and/or sensory‐based practices in preschools. Loomis (2018) suggests that universal implementation is essential for policymakers to support the continuous development of a trauma‐informed workforce, fostering relationships between parents and preschools, nurturing preschooler–teacher relationships, and ensuring access to targeted mental health services. However, trauma‐informed education models in schools encompass both individual (specific child‐focussed) implementations, targeting particular student needs and universal, school‐wide approaches (Chafouleas et al., 2015). This is of particular interest to occupational therapy due to the profession's integral role within learning and support teams in schools. Occupational therapists can play a key role in enabling early intervention, reducing strain on teachers, and addressing behavioural challenges, all of which contribute to creating more productive and inclusive learning environments for preschoolers (OTA, n.d.). Whereas there is ample evidence on trauma‐informed education for older children (Loomis, 2018; Purtle, 2020), there is a scarcity of evidence and frameworks for trauma‐informed preschools (Loomis, 2018). In the findings of our review, universal (whole class) sensory‐based practices were not necessarily linked to trauma‐informed approaches. Furthermore, Fraser et al. (2017) focussed on sensory‐based practices targeted at the individual level. Thus, a gap exists in the evidence base for both trauma‐informed and/or sensory‐based practices regarding the effectiveness of universal and individual implementation. This presents an opportunity for further research to inform practice for occupational therapists working at classroom and individual levels, as well as enhancing their role in consulting, collaborating, and upskilling other professionals, aiming to support participation in early childhood occupations (Campbell et al., 2023; World Federation of Occupational Therapists, 2016).

We found evaluation occurs at child, staff, and organisational levels, reflective of the scope of intervention approaches. The findings highlight complexities in evaluating children at an individual level, reflecting the various effects of trauma on their development (Enlow et al., 2012; National Scientific Council on the Developing Child, 2014), behaviour (Paolucci et al., 2001), social interactions (Jimenez et al., 2016), and academic outcomes (Perfect et al., 2016). Furthermore, conducting an evaluation of preschool educators and their organisations is reflective of the role quality preschool settings can have in supporting disadvantaged children (Bartlett & Smith, 2019; Næsby, 2021). Evidence suggests that policymakers should recognise the impact of trauma to support the evaluation of trauma‐informed practices (Loomis, 2018). Further, the current evidence focusses on short‐term outcomes of trauma‐informed and/or sensory‐based practices in preschools. Given the well‐established long‐term impacts of trauma on health and development (Alisic et al., 2014; Anda et al., 2006; Shonkoff & Phillips, 2000) and academic outcomes and behaviour (Jimenez et al., 2016; Milot et al., 2010), an opportunity exists for research to include follow‐up assessments. Occupational therapy can play a pivotal role in expanding the evidence base to be occupation‐centred, promoting its role in collaborative school‐based approaches, and contributing to the development of reliable assessment tools and interventions that foster meaningful participation in preschool settings (WFOT, 2016). Hence, it is crucial in both research and practice to consider the various effects of trauma and the support systems available when evaluating trauma‐informed and/or sensory‐based practices.

### 4.1 Limitations

Whereas this review employed a rigorous scoping review methodology, specific limitations exist. Potential biases or methodological weaknesses of included studies were not investigated (PRISMA‐ScR; Tricco et al., 2018). The review was restricted to primary studies published in English to evaluate current knowledge. Consequently, pertinent articles concerning trauma‐informed or sensory‐based practices, such as those from grey literature or articles published in other languages, may have been missed.

## 5 CONCLUSION

This scoping review found a small number (n = 11) of studies exploring trauma‐informed practices and even fewer on sensory‐based practices (n = 6) or the combination of both (n = 1). The lack of evidence for trauma‐informed and/or sensory‐based practices presents a challenge for occupational therapists working in preschool settings. This scoping review identified gaps in the current evidence base. There is some evidence for sensory‐based practices as a component of trauma treatment for children; however, more research is needed to establish practice efficacy to guide implementation in preschool settings. Future studies should consider the multifaceted effects of trauma on child outcomes, including its effects on sensory processing, to investigate how trauma‐informed and sensory‐based practices influence these outcomes in the preschool population. Given occupational therapy's broad role in both direct and embedded interventions in preschool settings, it is crucial to generate more evidence to inform and strengthen occupational therapy practice. Furthermore, future research should include follow‐up evaluations to evaluate long‐term impacts. Drawing from the review, we offer recommendations for an occupational therapy‐focussed research agenda with the intention to increase the evidence base to better inform evidence‐based practice.

## AUTHOR CONTRIBUTIONS

All authors were involved in the design of the scoping review, which was conceptualised by KR. RH, KPR, KH and KR were involved in title and abstract screening and RH and KR were involved in the full text review. RH manually searched reference lists of included studies. RH developed the data extraction template following team discussions and extracted the data for analysis. KPR checked 15% of the extracted data for consistency. RH, KPR, KH and KR reviewed the data extraction. RH led the analysis and summary of the results, under supervision of KR. RH prepared the draft manuscript with editing and revision by KPR, KH and KR. All authors approved the final manuscript.

## CONFLICT OF INTEREST STATEMENT

The authors have no conflict of interest to declare.

## Data Availability

The data that support the findings of this study are available from the corresponding author upon reasonable request.
